# Reliability of two methods for identifying the timing of medium latency responses in subjects with and without chronic ankle instability

**DOI:** 10.1038/s41598-019-40073-z

**Published:** 2019-02-28

**Authors:** Andreia S. P. Sousa, Isabel Valente, Ana Pinto, Rubim Santos

**Affiliations:** 1 Área Científica de Fisioterapia, Escola Superior de Saúde do Porto, Politécnico do Porto, Centro de Investigação em Reabilitação - Centro de Estudos de Movimento e Atividade Humana, Rua Dr. António Bernardino de Almeida, 400, 4200 - 072 Porto, Portugal; 2 Área Científica de Física, Escola Superior de Saúde do Porto, Politécnico do Porto, Centro de Investigação em Reabilitação - Centro de Estudos de Movimento e Atividade Humana, Rua Dr. António Bernardino de Almeida, 400, 4200 - 072 Porto, Portugal

## Abstract

This study aims to: (1) to compare 2 methods of assessing the timing of medium latency responses (MLR), in regard to intrasession reliability and mean values, in subjects with and without chronic ankle instability (CAI), and (2) to analyze the influence of CAI in timing of MLR and in its reliability. Thirty six athletes with (16) and without (20) CAI participated. Bilateral electromyography of peroneus longus (PL), peroneus brevis (PB), tibialis anterior (TA) and soleus (SOL) muscles was collected during a unilateral sudden inversion perturbation to assess the timing of MLR onset, in both standing and perturbed positions, through a baseline-based method and a peak-response-based method. The group without CAI presented higher relative reliability of SOL and peroneal muscles MLR with the peak response-based method than with the baseline-based method. Compared with the group without CAI and in both methods, the group with CAI presented a delayed and less reliable TA MLR, as well decreased coefficient variation of PL MLR in the uninjured limb. In conclusion, regardless of the method subjects with CAI present delayed and less reliable TA MLR while in subjects without CAI the peak response-based method provides higher reliability.

## Introduction

Motor control for even simple tasks is a plastic process that undergoes constant review and modification based upon the integration and analysis of sensory input, efferent motor commands, and resultant movements^1^. Proprioceptive information stemming from joint and muscle receptors plays an integral role in this process^1^. Among the responses involved in compensatory postural adjustments, the medium latency responses (MLR) appear to have a crucial role in the control of perturbations^2–4^. These are mediated by group II afferents through an oligosynaptic spinal pathway^5^, and possibly via group Ib afferents^6^. The evidence demonstrating the importance of muscle spindle type II and Ib force-sensitive fibers in the control of bipedal human stance^6–11^ probably by their strong dependence on the “postural set”^2^ support the high stabilizing effect of MLR during perturbations of stance^2^.

Based on the exposed, it can hypothesised that MLR are impaired in cases of chronic ankle instability (CAI). This hypothesis is strengthened by the evidence demonstrating that MLR can be modulated separately from the previously short latency responses (SLR)^5^ and the recently demonstrated reduced input from Ib afferents in both injured and uninjured limbs of athletes with CAI^12^. Recent studies have corroborated the theorized desensitization of group II fibers^13^ in cases of CAI by demonstrating that the magnitude modulation of tibialis anterior (TA) MLR is bilaterally impaired while assuming a support position and a perturbation was applied on the contralateral limb in cases of CAI^14,15^. These could lead to increased risk of contralateral ankle sprain in sudden inversion perturbations considering the role of the support limb in accelerating the centre of pressure in the direction of the support limb to dampen the contralateral ankle sprain mechanism^16^. Whereas a delayed onset of SLR of TA and soleus (SOL) muscles was demonstrated to occur in the support limb^15^, according to our knowledge, no study has evaluated the timing MLR in cases of CAI in this condition.

The difficulty of assessing the timing of MLR onset could explain the lack of studies regarding this variable. In fact, because the EMG may not return to baseline levels prior to the MLR response, the timing of onset of these responses could be difficult to assess. However, the evaluation of the timing of MLR after the reduction or disappearance of SLR, in other words, the timing of variation of MLR in relation to SLR could be a way of assessing the beginning of functional automatic postural responses in relation to the non-functional, spinal-mediated SLR^17^. In this sense, in the remaining document the concept of timing of MLR should be interpreted as the timing of variation of MLR in relation to SLR.

Various methods to determine the onset of the electromyographic activity which occurs in response to a stimulus have been discussed in the literature. Due to the stochastic characteristic of the surface electromyogram, onset detection is a challenging task^18^. Simple threshold-based methods (baseline-based methods) are very popular because of their intuitive and easy implementable structure^18^. Studies about the timing of peroneus SLR using this kind of method have demonstrated moderate to good reliability in healthy subjects^19–21^ and high reliability in subjects with CAI^21,22^. Based on these findings, high values of reliability would be expected with baseline-based method to assess the timing of MLR. Previous studies have used this method for the detection of the timing of MLR, however the reliability wasn’t evaluated^10,23^. Because the detection behaviour of simple threshold-based methods strongly depends on surface eletromyography parameters, such as onset rise time, signal-to-noise ratio or background activity level^18^, the reliability of this method in the detection of MLR timing could be compromised as MLR are preceded or even overlapped by SLR. An alternative method, based on the evidence that background electromyography activity measured before an external perturbation ranges between 2 and 5% of the size of SLR^24^, peak SLR response-based method, has been previously adopted^24,25^. However, despite some authors have used the timing of peak of MLR to quantify the timing of MLR^26^, according to our knowledge no study has used the peak response based method to assess the timing of MLR in response to an external perturbation.

Given the importance of MLR in stance postural stability, it is important to select a reliable method to assess the timing of this motor control variable, as measurement errors can seriously affect statistical analysis and interpretation^27^. Since reliability of measurement tools can be population^28^ and task specific, studies with the purpose of investigating relative and absolute reliability should be analyzed in healthy subjects, but also in subjects with postural control deregulation, such as subjects with CAI. Such knowledge has the potential to provide a foundation for answering research questions about the most reliable method to assess the timing of MLR in dysfunctional and non-dysfunctional conditions, and to assess motor control in those conditions. From a clinical point of view, this study contributes to establish how outcomes of interventions can be quantified to assess postural control as clinicians need measurement tools that show responsiveness and are able to detect minimal changes in performance^28^.

Based on the exposed, the aim of the current study was to compare the instrasession reliability between trials and the mean values of the timing of ankle MLR bilaterally in response to an unilateral simulated ankle sprain mechanism obtained by 2 methods (baseline-based vs. peak response-based methods) in healthy subjects and subjects with CAI. To assess the variability of each method, the intrasession reliability was calculated for MLR of ankle muscles in both standing and perturbed limbs considering the importance of both limbs in damping an unilateral ankle sprain mechanism^16^. Based on the evidence that the timing of ankle muscles is a reliable measure of the polysynaptic reflex to a sudden inversion stress^29^ and that the EMG may not return to baseline levels prior to the MLR response, it can be hypothesised, regardless of the limb position and group, differences between methods with higher levels of intrasession reliability with the peak response-based method. As a secondary aim, this study aimed to compare MLR between healthy subjects and subjects with CAI in terms of mean values as well in terms of intrasession reliability. Based on the previously exposed delayed onset timings of MLR are expected in subjects with CAI in both perturbed and standing positions.

## Methods

### Design

Cross sectional study.

### Participants

Sixteen athletes with unilateral CAI and twenty uninjured athletes participated in this study (Table 1). Participants assigned to the CAI group met the criteria set by the International Ankle Consortium^30^. For inclusion in the CAI group, subjects had to meet the following criteria: (1) history of at least one significant unilateral ankle sprain; (2) the initial sprain must have occurred at least 12 months prior to enrolment in the study; (3) at least one ankle sprain was associated with inflammatory symptoms; (4) at least one ankle sprain created at least one day of interruption of desired physical activity; (5) the most recent injury must have occurred more than three months prior to enrolment in the study; and (6) history of the previously injured ankle joint “giving way” and/or recurrent sprain and/or “feelings of instability”. To meet this last criterion, individuals must have answered “yes” to question 1 (“Have you ever sprained an ankle?”) along with “yes” to at least four questions related to perceived ankle instability and giving-way episodes: ‘(2) “Does your ankle ever feel unstable while walking on a flat surface?” (3) “Does your ankle ever feel unstable while walking on uneven ground?” (4) “Does your ankle ever feel unstable during recreational or sport activity?” (5) “Does your ankle ever feel unstable while going up stairs?” (6) “Does your ankle ever feel unstable while going down stairs?”. The CAI group included subjects presenting mechanical ankle instabiliy and/or functional ankle instability. Subjects were considered to have mechanical ankle instability if they presented one or more of the following conditions: 1) presence of pain or changes in talocrural joint mobility higher than 3 mm in the anterior drawer manual stress test (assessed using a triaxial accelerometer), compared to the uninjured side; and/or 2) talar tilt higher than 7° together with a difference higher than 0° in relation to the contralateral (uninjured) ankle (assessed using an electrogoniometer). The exclusion criteria for the CAI group met the criteria set by the International Ankle Consortium^30^ and included: (1) history of previous surgeries to the musculoskeletal structures in either limb of the lower extremity; (2) history of fracture in either limb of the lower extremity requiring realignment; (3) acute injury to musculoskeletal structures of other joints of the lower extremity in the previous three months, which impacted joint integrity and function resulting in at least one day of interruption of desired physical activity; and (4) history of bilateral ankle sprain.

**Table 1:** Mean and standard deviation (SD) values of age, height and weight subjects with and without chronic instability (CAI).

Variables	Mean (SD)		p-value
	Without CAI	With CAI	
Age (years)	21.8 (2.21)	20.5 (2.65)	0.940
Height (m)	1.78 (0.09)	1.76 (0.09)	0.800
Body weight (Kg)	73.8 (11.5)	69.6 (11.59)	0.248
Classification of CAI	—	FAI, n = 14 MAI, n = 10	—
Number of previous ankle sprains	—	3.1 (1.55)	—
Frequency of giving way	—	Rarely, n = 7 Frequently, n = 6 Often, n = 3	—
Severity of ankle sprain	—	Severe ankle sprain, n = 2 Moderate ankle sprain, n = 14	—
Time since last sprain (months)	—	10.5 (3.96)	—
	*n* = *20*	*n* = 16	

Healthy control participants were selected according to the same exclusion criteria applied to the CAI group and were also excluded if they had history of ankle sprain. All volunteers were athletes practicing sports with high risk of ankle sprain, including soccer, basketball, volleyball and handball. Prior to testing, subjects were asked to identify the dominant limb, which was described as the leg which they would use to kick a ball. As no differences were observed between the dominant and the non-dominant limb of healthy subjects in a previous study that used a similar protocol to the one used in the present study^16^, in the healthy control group this limb was selected for evaluation. In the CAI group both limbs were evaluated.

The study was approved by the local ethics committee (Escola Superior de Saúde do Porto (1719/2014)) and was implemented according to the Declaration of Helsinki. All subjects gave their written informed consent for study participation participate.

### Instruments

The activity of the agonist muscles for active ankle stability, peroneus longus (PL), peroneus brevis (PB), TA and SOL muscles, was assessed by electromyography. The electromyographic signal of these muscles was monitored using a bioPLUX research wireless signal acquisition system. The signals were collected at a sampling frequency of 1000 Hz and were preamplified in each electrode and then fed into a differential amplifier with an adjustable gain setting (25–500 Hz; common-mode rejection ratio: 110 dB at 50 Hz, input impedance of 100 MΩ and gain of 1000). Self-adhesive silver chloride electromyographic electrodes were used in a bipolar configuration with a distance of 20 mm between detection surface centers. The skin impedance was measured with an electrode impedance checker. The electromyography and force platform signals were analysed with the Acqknowledge software.

The Ankle Instability Instrument was designed to classify patients with functional ankle instability and has been shown to be a reliable and valid tool^31^. The instrument presents high values of test-retest reliability (ICC = 0.95). lnternal consistency reliability estimates (alpha coefficients) for each factor and the total measure ranged from 0.74 to 0.83.

A tilt platform was used to force 30° of subtalar joint inversion. The platform included two movable plates (trapdoors) so that either foot could be tilted independently, thus removing any subject anticipatory effect. A triaxial accelerometer (bioPLUX research) connected to a wireless signal acquisition system was placed in each movable plate to detect the onset of the tilt mechanism (first deflection of the accelerometer signal). For safety reasons, the tilt platform was surrounded by a handrail to the front and both sides of the subject and an adhesive, non-slip material was placed to create a footpath and to prevent slipping when the trapdoors were dropped.

### Procedures

The skin surface of the selected muscles mid-belly and of the patella was prepared (shaved, dead skin cells and non-conductor elements were removed with alcohol and with an abrasive pad) to reduce the electrical resistance to less than 5000 Ω. The placement of electrodes for recording EMG activity was based on recommendations reported in the literature^32^. For the TA the electrode was placed in the 1/3 on the line between the tip of the head of the fibula and the tip of the medial malleolus. For the SOL the electrode was placed 2 cm distal to the lower border of the medial gastrocnemius muscle belly and 2 cm medial to the posterior midline of the leg. For PB the electrodes were placed anterior to the tendon of the PL at 25% of the line from the tip of the lateral malleolus to the fibula-head. For the PL the electrodes were placed at 25% on the line between the tip of the head of the fibula to the tip of the lateral malleolus.

**Figure 1:**
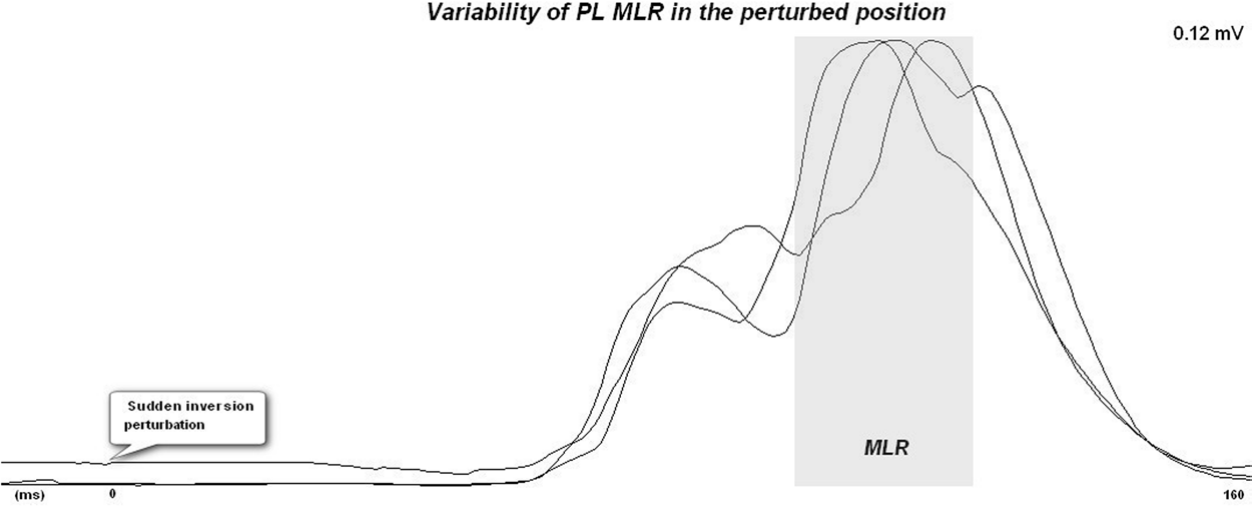
Representation of the variability obtained between trials for the PL muscle in the perturbed position. In this figure is can be observed low variability of the MLR.

**Figure 2:**
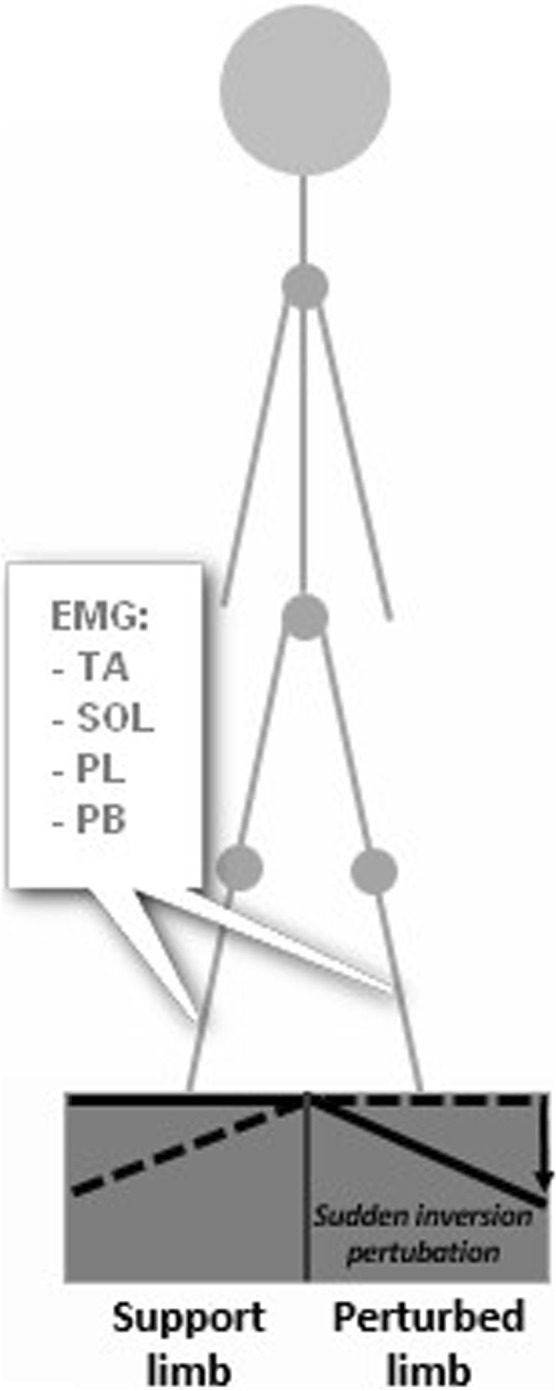
Representation of the set up adopted. The figure illustrates a trial where a sudden ankle inversion was applied in the right limb. The EMG signals of TA, SOL, PL and PB from support (left) limb and perturbed (right) limb were collected.

All individuals were asked to stand quietly with the support base aligned at shoulder width with one foot in each trapdoor, keeping their arms by their sides, and to focus on a target 2 meters away and at eye level during 30 seconds. The weight of participants was used to generate the speed. To guarantee the same velocity of inversion between trials, the individuals were instructed to ensure equal weight distribution between the two limbs. One limb at a time was randomly exposed to the sudden inversion perturbation and was identified during analysis of each trial as the perturbed limb. The limb which was not exposed to the simulated ankle sprain in each trial was identified as the support limb. Each limb was exposed to the sudden inversion perturbation three times in a random order. Previous studies have demonstrated high reliability values of ankle muscles latency in response to a forced inversion perturbation by using six trials^20^. However, since in our pilot study we have found that the reliability values obtained from three or six valid trials were very similar in both subjects with and without CAI we decided to perform only three trials (Fig. 1). In each trial the trapdoor was randomly released by pushing a foot switch not visible for the subject. The subjects did not know the side nor the time of application of the perturbation. In the CAI group, the electromyographic signal was collected from both limbs (injured and uninjured limbs) and both where evaluated as support and perturbed limbs. In the control group, only the non-dominant limb was monitored in the standing position (3 trials) and in the perturbed position (3 trials). Upon release, the platform fell down through an arch of 30° which was predetermined by a mechanical stop leading to ankle subtalar inversion (Fig. 2). Rest periods of 60 seconds were provided between trials, during which the subjects sat down while maintaining the foot position.

The electromyographic signals were filtered using a zero-lag, second-order Butterworth filter with an effective bandpass of 20 to 450 Hz. The signal was posteriorly full wave rectified and first order lowpass filtered (20 Hz)^33^. Two methods were used to identify the timing of MLR: (1) a baseline-based method and (2) peak response-based method. In both methods, identification was achieved using a computer program and visual inspection.

#### Baseline-Based Method

Considering that the ratio between SLR and MLR can be lower or higher than 1^34^, the latency for MLR of PL, PB, TA and SOL muscles of each limb (support and perturbed positions) was defined according to criteria: 1) as the instant when its EMG amplitude was higher than the mean value plus 3 standard deviations (SD), or 2) as the instant when its EMG amplitude was lower than the mean value minus 3 standard deviations (SD)^35^. The mean value was assessed in the first 20 ms after the beginning of SLR, using a combination of computational algorithms and visual inspection. The value was considered valid when it was maintained above or below the threshold at least 20 ms and had returned to values close to baseline values between SLR and MLR^5,9,23,25,36–39^. When values did not return close to baseline values but a clearly second peak was identify, the value was also considered valid since MLR can overlap SLR^34^. The baseline values were obtained in the interval between -450 and -500 ms in relation to the first deflection of the accelerometer signal (T0). The latency for SLR of PL, PB, TA and SOL muscles was defined as the instant when its EMG amplitude was higher than the mean of its baseline value plus 3 standard deviations (SD), measured from − 500 to − 450 ms in relation to T0. To be considered a SLR, the signal must be above the threshold at least 10 ms, since it has been stablished the minimum value for the duration of these responses^38,40–42^. When this criteria wasn’t verified but the response was followed by a MLR identified as a second peak, the response was also considered as SLR considering that in individuals with CAI MLR can overlap SLR^43^.

#### Peak response-based method

The latency of MLR was defined as the instant when was obtained a value equal or higher than 5% of the second peak of compensatory responses. The peak value was calculated in the interval from 20 ms to 40 ms in relation to the beginning of SLR. The timing of MLR was considered valid when it was maintained above the threshold at least 20 ms and had returned to values close to baseline values between SLR and MLR^9,37–41^. When values did not returned close to baseline values but a clearly second peak was identify the value was also considered valid since MLR can overlap SLR^34^.

**Figure 3:**
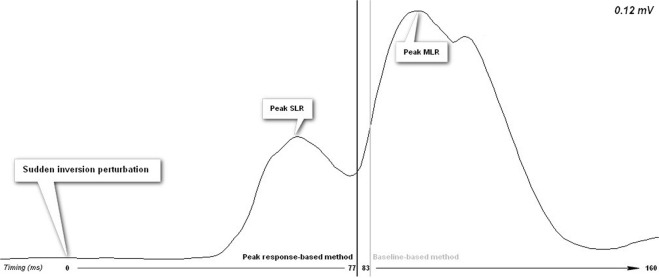
Representation of short latency (SLR) and medium latency (MLR) responses of PL muscle of the limb exposed to a sudden ankle inversion in a single trial of data. The vertical black line represents the timing of MLR calculated with the peak response-based method and the grey vertical line represents a later timing of MLR calculated with baseline-based method.

The threshold’s selection was adapted from methods used on previous studies that have used the same criterion for other biomechanical variables, and on the fact that it provided a good agreement with visual inspection^24,25^. A representation of the timings of MLR obtained with each method for one trial of a participant is provided in Fig. 3.

### Data analysis

The acquired data were analysed using the Statistic Package Social Science (SPSS) software from IBM Company (USA). Reliability measures of the timing of MLR assessed from each method were calculated for athletes with and without CAI. The Intraclass correlation coefficient (ICC2,1) with a 95% confidence interval (CI) was used because it considers random effects over time and expresses relative reliability of the timing of MLR obtained with each method^27^. Specifically, a 2-way ANOVA model with a random subject effect was used to estimate the intrasession reliability. The following range of reliability coefficients were used to report the degree of reliability: 0.00–0.25 = little, if any correlation; 0.26–0.49 = low correlation; 0.50–0.69 = moderate correlation; 0.70–0.89 = high correlation; and 0.90–1.00 = very high correlation^44^. The coefficient of variation (CV) was used to express absolute reliability and was calculated per subject, by dividing SD by the mean of 3 trials.

The reliability and values of timing of MLR were compared regarding the method (baseline-based method vs peak response based method) and the group (group with CAI vs group without CAI). For both comparisons, the limb position (standing vs perturbed) was considered as well the side of CAI in the group with CAI (injured vs uninjured limb). Shapiro–Wilk test results and histogram analysis have shown that data were normally distributed. The statistical difference between ICCs of each method (baseline-based method vs peak response based method in each group) but also between groups (group with vs group without CAI in each method) was evaluated through the application of Fisher’s Z transformation, with significance determined with the t statistic. The paired samples t test was used to compare the CV and timing of MLR values obtained between methods (baseline-based method vs peak response based method in each group). The independent samples t test was used to compare mean values of timing of MLR and CV values between athletes with and without CAI (group with vs group without CAI in each method). A 0.05 significance level was used for inferential analysis.

## Results

### Baseline – based method vs. Peak response – based method

#### Group without CAI

In the standing position high correlations were observed for the timing of MLR of all muscles when the peak response – based method was used. When baseline - based method was used, high correlations were only observed for TA MLR (Table 2). When both methods were compared, increased ICC values were observed in timing of MLR of SOL (p = 0.001), PL (p = 0.014) and PB (p = 0.007) muscles and later onset timings were observed in SOL MLR with peak response – based method compared to baseline – based method (p = 0.031), Table 2.

**Table 2:** Mean values and standard deviation (SD) of medium latency responses (MLR) (ms) and coefficient of variation (CV) (%) and intraclass correlation coefficient (ICC) (95% confidence interval (CI)) obtained for tibialis anterior (TA), soleus (SOL), peroneal longus (PL) and peroneal brevis (PB) muscles in the standing position in subjects with and without chronic ankle instability (CAI). P values obtained from comparisons between subjects with and without CAI are presented.

			Uninjured limb						Injured limb					
			Mean (SD)	p	CV C%	p	ICC (95% CI)	p	Mean (SD)	p	CV %	p	ICC (95% CI)	p
Baseline-based method	**TA**	*Without CAI*	69.4 (2.75)	0.010**	29.0	0.169	0.82 (0.49, 0.95)	0.012*	—					0.241
		*With CAI*	90.2 (1.42)		18.2		0.33 (−0.97, 0.82)		84.2 (1.86)	0.086	15.5	0.058	0.89 (0.73, 0.96)	
	**SOL**	*Without CAI*	78.8 (2.27)	0.611	33.1	0.677	0.10 (−0.98, 0.64)	0.016*	—					0.147
		*With CAI*	92.2 (1.89)		31.0		0.71 (0.03, 0.94)		72.7 (1.80)	0.433	34.9	0.891	0.45 (-0.26, 0.80)	
	PL	*Without CAI*	81.9 (2.46)	0.259	30.0	0.040*	0.49 (−0.49, 0.86)	0.168	—					0.172
		*With CAI*	88.4 (1.32)		14.7		0.71 (0.03, 0.94)		82.3 (1.95)	0.611	22.2	0.351	0.71 (0.31, 0.90)	
	PB	*Without CAI*	90.1 (3.11)	0.884	24.1	0.090	0.37 (-0.96, 0.85)	0.001**	—					0.18
		*With CAI*	91.6 (1.66)		15.7		0.92 (0.76, 0.98)		85.2 (2.27)	0.962	19.1	0.403	0.62 (0.10, 0.86)	
Peak response based method	**TA**	*Without CAI*	73.2 (2.78)	0.019*	27.3	0.294	0.89 (0.73, 0.96)	0.006**	—					0.501
		*With CAI*	90.5 (0.94)		18.8		0.46 (−0.67, 0.87)		83.7 (1.88)	0.219	17.9	0.177	0.89 (0.74, 0.96)	
	**SOL**	*Without CAI*	93.0 (0.85)	0.258	24.1	0.645	0.84 (0.61, 0.94)	0.305	—					0.495
		*With CAI*	94.8 (1.29)		28.5		0.78 (0.30, 0.94)		75.9 (2.88)	0.037	23.5	0.942	0.79 (0.50, 0.92)	
	PL	*Without CAI*	84.3 (2.50)	0.158	26.1	0.041*	0.73 (0.33, 0.90)	0.157	—					0.157
		*With CAI*	96.0 (1.19)		21.1		0.86 (0.53, 0.97)		80.5 (1.73)	0.638	21.1	0.519	0.53 (-0.11, 0.83)	
	PB	*Without CAI*	84.9 (2.50)	0.552	27.1	0.002**	0.84 (0.58, 0.95)	0.277	—					0.277
		*With CAI*	97.3 (1.16)		23.8		0.78 (0.30, 0.94)		85.3 (2.12)	0.963	23.8	0.649	0.90 (0.74, 0.96)	

*p < 0.05, ** < 0.01.

In the perturbed limb an high correlation was only observed for SOL MLR with both methods (Table 3). While moderate correlations were found for all the other muscles with the peak response – based method, when baseline-based method was used a moderate correlation was only observed in PL muscle (Table 3). No statistically significant differences were observed between methods regarding their mean and reliability values for this position.

**Table 3:** Mean values and standard deviation (SD) of medium latency responses (MLR) (ms) and coefficient of variation (CV) (%) and ICC (95% confidence interval (CI)) obtained for tibialis anterior (TA), soleus (SOL), peroneal longus (PL) and peroneal brevis (PB) muscles in the perturbed position in subjects with and without chronic ankle instability (CAI). P values obtained from comparisons between subjects with and without CAI are presented.

			Uninjured limb						Injured limb					
			Mean (SD)	p	CV C%	p	ICC (95% CI)	p	Mean (SD)	p	CV %	p	ICC (95% CI)	p
Baseline-based method	**TA**	*Without CAI*	65.8 (23.4	0.238	28.6	0.259	0.33 (−0.51, 0.74)	0.138	—					0.316
		*With CAI*	75.0 (17.86)		21.6		0.63 (0.02, 0.88)		64.7 (18.37)	0.876	35.2	0.361	0.48 (−0.21, 0.80)	
	**SOL**	*Without CAI*	79.4 (31.70)	0.421	29.5	0.268	0.72 (0.40, 0.88)	0.083	—					0.397
		*With CAI*	88.4 (29.32)		21.2		0.89 (0.69, 0.93)		73.7 (15.96)	0.542	23.1	0.302	0.76 (0.44, 0.91)	
	PL	*Without CAI*	73.5 (23.53)	0.260	30.6	0.512	0.59 (0.13, 0.82)	0.114	—					0.229
		*With CAI*	83.9 (28.12)		25.2		0.81 (0.43, 0.95)		72.5 (21.07)	0.893	21.7	0.186	0.73 (0.37, 0.90)	
	PB	*Without CAI*	73.5 (25.63)	0.878	26.8	0.137	0.39 (−0.28, 0.74)	0.001**	—					0.360
		*With CAI*	78.0 (26.31)		16.6		0.92 (0.76, 0.98)		77.1 (14.98)	0.638	24.2	0.679	0.50 (-0.16, 0.81)	
Peak response based method	**TA**	*Without CAI*	69.1 (24.56)	0.341	30.7	0.135	0.65 (0.15, 0.88)	0.382	—					0.435
		*With CAI*	76.7 (18.12)		19.3		0.71 (0.24, 0.91)		66.3 (18.17)	0.710	33.8	0.671	0.69 (0.25, 0.89)	
	**SOL**	*Without CAI*	83.8 (35.99)	0.504	29.7	0.218	0.83 (0.58, 0.94)	0.329	—					0.444
		*With CAI*	91.4 (29.71)		20.4		0.87 (0.65, 0.96)		78.9 (20.65)	0.669	23.8	0.354	0.81 (0.56, 0.93)	
	PL	*Without CAI*	77.0 (26.44)	0.662	28.0	0.818	0.52 (−0.18, 0.83)	0.162	—					0.165
		*With CAI*	80.9 (22.79)		26.1		0.73 (0.26, 0.92)		68.8 (19.75)	0.325	22.7	0.368	0.73 (0.38, 0.90)	
	PB	*Without CAI*	77.8 (24.79)	0.462	33.3	0.130	0.56 (−0.08, 0.85)	0.018*	—					0.286
		*With CAI*	79.4 (26.60)		17.1		0.89 (0.66, 0.97)		77.1 (14.24)	0.920	28.6	0.592	0.40 (−0.39, 0.77)	

*p < 0.05, ** < 0.01.

#### Group with CAI

High correlations were observed in SOL, PB and PL MLR in the uninjured limb in standing position with both methods (Table 2). In the injured limb, in the standing position, high correlations were observed in TA, SOL and PB MLR with the peak response – based method and in the SOL and PL MLR with baseline-based method (Table 2). In the perturbed position, high correlations were observed in the uninjured limb for SOL, PL and PB MLR for both methods (Table 3). High ICC values were found for TA MLR only with the peak response – based method (Table 3). In the injured limb, in perturbed position, high correlations were only observed for SOL and PL MLR with both methods (Table 3). Despite a tendency to higher ICC values in the peak response – based method (Table 2), no statistically significant differences were observed in reliability variables as well in timing of MLR between methods in subjects with CAI in both standing and perturbed positions.

### Group without CAI vs. Group with CAI

Later onset timing of TA MLR, decreased TA MLR ICC values and decreased CV PL MLR values were observed in the uninjured limb of CAI group compared to the group without CAI with both peak response – based and baseline – based methods in the standing position (Table 2). Decreased CV values were also observed in PB MLR of the uninjured limb of CAI group in the standing position, but only with peak response based method (Table 2). Increased ICC values were found for PB and SOL MLR in the uninjured limb of CAI group with the baseline – based method (Table 2) and an earlier onset timing of SOL MLR was observed in the injured limb of CAI group with the peak response based method in the standing position (Table 2).

In the perturbed position, differences between groups were only observed in the ICC of PB MLR (Table 3). Higher ICC values were observed in the uninjured limb of CAI group compared to the group without CAI. (Table 3).

## Discussion

In the present study the timing of MLR in response to an unilateral forced inversion movement was assessed by two methods in both injured and uninjured limb of subjects with CAI and in the nondominant limb of subjects without CAI. Each limb was evaluated while assuming a standing and perturbed position.

### Baseline-based method vs peak response based method

The values obtained with both methods in participants without CAI agree with previously one. The values obtained for TA MLR in support (69.4–73.2 ms) and perturbed (65.8–69.1 ms) positions agree with the one obtained by Corna *et al*.^45^ (67–71 ms) and by Toft *et al*.^33^ (69–80 ms). The MLR of SOL in support (93.0 ms) and perturbed (83.8 ms) positions with the peak response-based method agree with the 91 ms presented by Grüneberg *et al*.^46^ through visual inspection, while the values obtained with baseline-based method (support (78.8 ms), perturbed (79.4 ms) positions) are more close to the 78 ms proposed for the peak of MLR^5^. For the peroneal muscles, our findings demonstrate for the perturbed position earlier onset timings (PL (73.5–77.0 ms), PB (73.5–77.8 ms)) than the previously demonstrated (85 ms^47^ for PL and 89.4 ms and 90.0 ms for PB^46^. The values obtained for the support position (PL (81.9–84.3 ms), PB (90.1–84.9 ms)) are more close to the previously obtained. These findings together the non-existence of statistically significant differences between methods regarding the timing of MLR (with the exception of SOL) seem to demonstrate that both methods can be used to identify the onset timing of MLR of ankle muscles. However, differences between methods were obtained as to intrassession reliability. Globally higher intrassession reliability values were obtained with the peak response-based method compare to baseline-based method in participants with and without CAI. While the baseline based method was based on the mean of the first 20 ms after da beginning of short latency responses, the peak response – based method relies on the peak of medium latency responses. These findings seem to demonstrate higher stability in the peak MLR than in the mean and standard deviation values of SLR. When both methods were compared as to the relative reliability, statistically significant differences were only observed in the group without CAI in the standing position in MLR of SOL, PL and PB muscles. These findings reveal that despite increased relative reliability was found with the peak response – based method in both groups, the differences are more pronounced in the group without CAI. Some authors recommend a minimum ICC value of 0.70 for research purposes^48^ while others defend that this value cannot be set in absolute terms and that should be taken into account the aim of the instrument under investigation^28^. In the present study both groups presented values higher or close to 0.70 in both positions with peak response – based methods for the majority of the muscles. The relative reliability is the degree to which individuals maintain their position in a sample with repeated measurements^49^. The results of the present study indicate that peak response based method fulfils this purpose. However, this variable is influenced highly by the range of measured values. When methods were compared as to absolute reliability, no differences were observed in both groups indicating that both methods are similar in the degree to which repeated measurements vary for individuals^49^.

### Group with CAI vs group without CAI

Later onset timing of TA MLR was observed in the uninjured limb of CAI group with both methods in a support position, associated to a tendency to later onset timing of MLR of all the other muscles in both positions when compared to participants without CAI. This results seem to demonstrate that both methods are capable of identifying postural control deregulation related to the timing of MLR. In fact, a delayed onset of TA MLR together with a decreased relative reliability observed in the uninjured limb in the standing position compared to the group without CAI was observed in both methods. A postural control deregulation in the uninjured limb in participants with unilateral history of ankle sprain has been recently demonstrated^14,15^. In the injured limb, earlier SOL MLR was found in the standing position but only with the peak response – based method. This difference is probably related to the fact that later onset timing of SOL MLR was observed in the peak response-based method compared to baseline-based method in the standing of limb of participants without CAI. The peak response – based method seems less sensible in determining the timing of SOL MLR in the standing position in subjects without CAI. It was interesting to note that for peroneus muscles high absolute reliability was found for PL and PB MLR in the uninjured limb in standing position and in the relative reliability of both limbs of CAI group in the perturbed position, all with both methods. SOL MLR of uninjured limb in standing position presented also increased relative variability with baseline – based method compared to the group without CAI. These findings reveal that subjects with CAI present more stereotyped patterns of postural responses mainly in peroneus muscle.

### Limitations

It should be noted that only three trials were performed, intrasession reliability studies involving more trials are required. The lack of mechanical variables like centre of pressure displacement is the major limitation of the present study since limits the interpretation of the differences between groups regarding the timing of MLR and its reliability. The non-evaluation of the level of disability of the CAI group limits the comparisons of the results obtained in the present study with the ones obtained in previous studies.

## Conclusion

The peak response – based method present higher intrasession reliability than baseline –based method in subjects with and without CAI. Subjects with CAI present bilateral delayed and decreased relative reliability of TA MLR in a standing position and increased relative and absolute intrasession reliability of MLR of peroneus muscles in both standing and perturbed positions.

## Key Points

- **Findings:** The peak response – based method present high intrasession reliability in detecting medium latency responses (MLR). Subjects with chronic ankle instability (CAI) present delayed tibialis anterior (TA) MLR in a support position.
- **Implications:** Both limbs should be integrated in the rehabilitation following unilateral ankle sprain since MLR are altered in both ipsilesional and contralesional limbs.
- **Caution:** It should be noted that the differences observed are valid for three trials. Future studies are required to confirm if this pattern is maintained with more trials since it will probably express higher representation of the response pattern.
